# Association Between Delivery Room Continuous Positive Airway Pressure and Neonatal Outcomes in Late Preterm and Term Infants: A Systematic Review and Meta‐Analysis

**DOI:** 10.1111/jpc.70516

**Published:** 2026-07-28

**Authors:** Yoxin Chin, Maclean Hill, Arun Sett, Niranjan Thomas, Abdul Razak

**Affiliations:** ^1^ Newborn Services Joan Kirner Women's and Children's Hospital Melbourne Victoria Australia; ^2^ Neonatal Research Murdoch Children's Research Institute Melbourne Victoria Australia; ^3^ Department of Obstetrics, Gynaecology and Newborn Health The University of Melbourne Melbourne Victoria Australia; ^4^ Department of Paediatrics Monash University Melbourne Victoria Australia; ^5^ Monash Newborn Monash Children's Hospital Melbourne Victoria Australia; ^6^ The Ritchie Centre Hudson Institute of Medical Research Melbourne Victoria Australia

**Keywords:** clinical trials, continuous positive airway pressure, delivery room, meta‐analysis, pneumothorax

## Abstract

**Objective:**

To explore the effects of delivery room continuous positive airway pressure (DR‐CPAP) in late preterm and term infants.

**Design:**

Systematic review and meta‐analysis.

**Data Sources:**

Cochrane Library, Embase, Emcare and MEDLINE were searched through March 26, 2025.

**Eligibility Criteria:**

Observational studies and randomised clinical trials (RCTs) comparing outcomes in infants receiving DR‐CPAP versus no DR‐CPAP.

**Methods:**

Meta‐analyses were conducted using a random‐effects model to calculate pooled odds ratios (ORs) and 95% confidence intervals (CIs). Risk of bias was assessed per Cochrane guidelines, and certainty of evidence was graded using the GRADE framework.

**Main Outcome:**

Pneumothorax.

**Results:**

Seven studies (five non‐randomised, two RCTs) involving 135 472 infants were included. In observational data, DR‐CPAP was associated with a significant increased risk of pneumothorax (unadjusted OR: 45.79; 95% CI: 31.47–66.63; *moderate certainty*; adjusted OR: 24.18; 95% CI: 2.32–251.63; *moderate certainty*). RCTs reported no pneumothorax events in both groups, preventing meta‐analytic estimation. DR‐CPAP was also associated with increased use of surfactant in observational studies (OR: 5.68; 95% CI: 2.97–10.86; *very low certainty*). For neonatal intensive care unit admissions, DR‐CPAP was associated with significantly higher odds in observational studies (OR: 14.56; 95% CI: 13.27–15.98; *moderate certainty*), but significantly lower odds in RCTs (OR: 0.24; 95% CI: 0.09–0.63; *very low certainty*).

**Conclusions:**

DR‐CPAP was associated with potential harm in observational studies, though confounding cannot be excluded. Evidence from RCTs remains limited, and is of very low certainty. Well‐designed, robust clinical studies are urgently needed to guide practice.

**Abbreviations**
BW: birth weight
CI: confidence interval
CPAP: continuous positive airway pressure
DR: delivery room
GA: gestational age
GRADE: grading of recommendations assessment, development, and evaluation
ILCOR: International Liaison Committee on Resuscitation
NICU: neonatal intensive care unit
OR: odds ratio
RCT: randomised controlled trial

## 1 Introduction

Continuous positive airway pressure (CPAP) in the delivery room is a cornerstone of respiratory support for preterm neonates, particularly those born extremely preterm (before 28 weeks' gestation), as endorsed by the International Liaison Committee on Resuscitation (ILCOR) [1]. In this population, applying a constant pressure at the end of expiration effectively stabilises functional residual capacity and supports work of breathing, reduces the need for mechanical ventilation [2, 3], and reduces complications like bronchopulmonary dysplasia [4, 5, 6]. However, the application of CPAP has increasingly extended to late preterm (34–36 weeks) and term (37 weeks and beyond) infants with respiratory distress, despite ILCOR guidelines noting insufficient evidence to support its routine use in this population [1]. This shift in practice prompts a closer examination of whether a uniform approach to using delivery room CPAP, originally tailored to the needs of extremely preterm infants, can be safely and effectively applied to infants born closer to or at term.

The physiology of late preterm and term infants differs significantly from that of their extremely preterm counterparts [5]. Lungs in late preterm and term infants are comparatively more developed and compliant, with greater alveolar stability, airway stability, and surfactant quantity and quality [7, 8]. While CPAP in extremely preterm infants compensates for developmentally immature physiology, its role in more mature lungs may not yield the same benefits. Due to the physiological maturity of the late preterm respiratory system, the application of CPAP based on evidence extrapolated from more immature infants may have unintended consequences [9, 10, 11]. Given these distinctions, the routine use of delivery room CPAP (DR‐CPAP) in late preterm and term infants warrants scrutiny to determine its appropriateness and safety [1]. Some reports have suggested an association with pneumothorax [9, 10, 11], a complication potentially linked to the application of positive pressure in more compliant lungs unaccustomed to such support. This systematic review and meta‐analysis aimed to evaluate the effects of DR‐CPAP in late preterm and term infants, exploring whether its routine use aligns with favourable outcomes or if it may, in fact, confer harm, such as an increased incidence of pneumothorax, thereby guiding evidence‐based refinements to neonatal resuscitation practices.

## 2 Methods

This systematic review and meta‐analysis was prospectively registered with Open Science Framework (OSF; registration DOI: https://osf.io/fj8pe/). It is conducted and reported as per the guidelines from preferred reporting items for systematic reviews and meta‐analyses (PRISMA) [12].

### 2.1 Search Strategy

A systematic search of the literature was conducted using appropriate pre‐specified search terms within the following databases: Cochrane Central Register of Controlled Trials, Excerpta Medica Database (Embase), Emcare, and Medical Literature Analysis and Retrieval System Online (MEDLINE) from inception through March 26, 2025. The detailed search strategy for each database is outlined in the Supporting Information. Additionally, the reference lists and citations of studies included in the systematic review were searched to identify further relevant studies.

### 2.2 Eligibility Criteria

*Type of studies*: Randomised controlled trials (RCTs) and observational studies evaluating one or more predefined outcomes of systematic review were eligible for inclusion in the study. Systematic reviews, descriptive studies, case series and case reports were excluded.

*Population*: The population of interest comprises late preterm (34–36 weeks' gestation) and term (≥ 37 weeks' gestation) infants. Studies with mixed gestational ages, including those < 34 weeks, were included if separate data for late preterm and term infants were provided.

*Exposure and Comparison*: The exposure of interest is the administration of CPAP in the delivery room to late preterm and term infants (≥ 34 weeks' completed gestation). The comparator group includes infants who did not receive CPAP but may have received other forms of support, such as supplemental oxygen or low‐flow oxygen therapy. Infants requiring higher levels of respiratory support than CPAP, such as non‐invasive ventilation or mechanical ventilation in the delivery room, were not considered part of the comparison group.

*Outcomes*: The following outcomes were evaluated in this systematic review:

1. *Primary outcome*: Pneumothorax, determined by any lung imaging technique, including chest x‐ray or lung ultrasound, at any time before discharge.
2. *Secondary outcomes*: Pneumothorax requiring intervention (needle thoracocentesis or intercostal drain), the need for intubation and ventilation (as defined by authors for any indication), surfactant administration (as defined by authors for any indication), duration of respiratory support (as defined by authors), pulmonary haemorrhage (as defined by authors), neonatal intensive care unit (NICU) admission (as defined by authors for any indication) and mortality. All outcomes assessed before discharge.

### 2.3 Data Extraction

Y.C. searched the databases as listed above with the help of an information specialist. Y.C. and M.H. screened the titles and abstracts using Covidence and the shortlisted studies were reviewed by Y.C. and M.H. to determine eligibility for inclusion based on screening form (see Supporting Information) [13]. Each of the study deemed eligible for inclusion was examined for information (study design, inclusion, key characteristics and outcomes) using a standardised data collection form. No blinding strategies were used. Any missing or unclear information was pursued by attempts at contacting the corresponding authors via email at least twice. Any discrepancies were resolved by a third author (N.T.). A final list of literature after the search was assimilated using an online reference management software EndNote 21 (Clarivate Analytics, Philadelphia, PA, USA).

### 2.4 Assessment of Risk of Bias

Risk of Bias in Non‐randomised studies of Exposures tool was used to assess the risk of bias for the observational studies [14]. This tool evaluates seven domains of bias: confounding, selection of participants, classification of exposures, deviations from intended exposures, missing data, measurement of outcomes and selection of reported results. Studies were classified as having low risk of bias, some concerns, high risk and very high risk. For RCTs, Cochrane Risk of Bias 2 tool was applied, covering five domains: randomisation process, deviations from intended interventions, missing outcome data, measurement of outcomes and selection of reported results [15]. The RCTs were classified as low, some or high concerns for risk of bias. The use of both these tools were performed by Y.C. and M.H. and any conflicts were resolved by N.T.

### 2.5 Data Synthesis and Statistical Analysis

Odds ratios (ORs) and 95% confidence intervals (CI) were calculated for each study that was included. Data synthesis involved pooling ORs with 95% CIs to estimate the effect of DR‐CPAP on outcomes, using a random‐effects model to account for anticipated heterogeneity across studies. Results given in risk ratios (RRs) were converted to ORs using the appropriate formula [16]. Analyses were performed separately for RCTs and observational studies, including unadjusted and adjusted data in observational studies, to explore the impact of confounding factors, with statistical analysis conducted using Review Manager version 5.3 (Cochrane Collaboration, Nordic Cochrane Center, Copenhagen, Denmark). For outcomes with significant differences, the absolute risk difference (ARD) and its 95% CIs were reported, along with the number needed to treat or harm and its 95% CI, to aid clinical interpretation. Cochrane Grading of Recommendations Assessment, Development, and Evaluation approach was used to assess the level of certainty of evidence [17].

### 2.6 Assessment of Heterogeneity

Pooled forest plots were qualitatively evaluated to assess heterogeneity. Statistical heterogeneity was quantified using *I* ^2^ values (derived from the chi‐squared *Q*‐statistic) in the meta‐analysis, with significant heterogeneity defined a *p*‐value for chi‐squared < 0.10. Upon detecting significant statistical heterogeneity, data were reviewed for errors or inconsistencies during extraction and entry. This was assessed independently by two authors (Y.C. and M.H.) and any discrepancies were resolved with a third author (A.R.). Subgroup analyses, if data were available, were conducted separately for late preterm infants (34–36 weeks' gestation) and term infants (≥ 37 weeks' gestation) to explore sources of heterogeneity. No additional strategies, such as meta‐regression, were planned a priori if subgroup did not resolve heterogeneity.

## 3 Results

### 3.1 Literature Search and Study Selection

A total of 1851 records were identified through the literature search. After removing 730 duplicates, 1121 records remained for title and abstract screening, of which 1099 were excluded. Twenty‐two full‐text articles were assessed for eligibility, with 18 excluded (reasons provided in the Supporting Information). An additional four reports were identified through other sources, of which two were excluded. In total, six reports were included in the review and meta‐analysis; one report contained two distinct studies, resulting in seven included studies (two randomised controlled trials and five observational studies). This process is presented in the PRISMA flow diagram (Figure 1).

**FIGURE 1:**
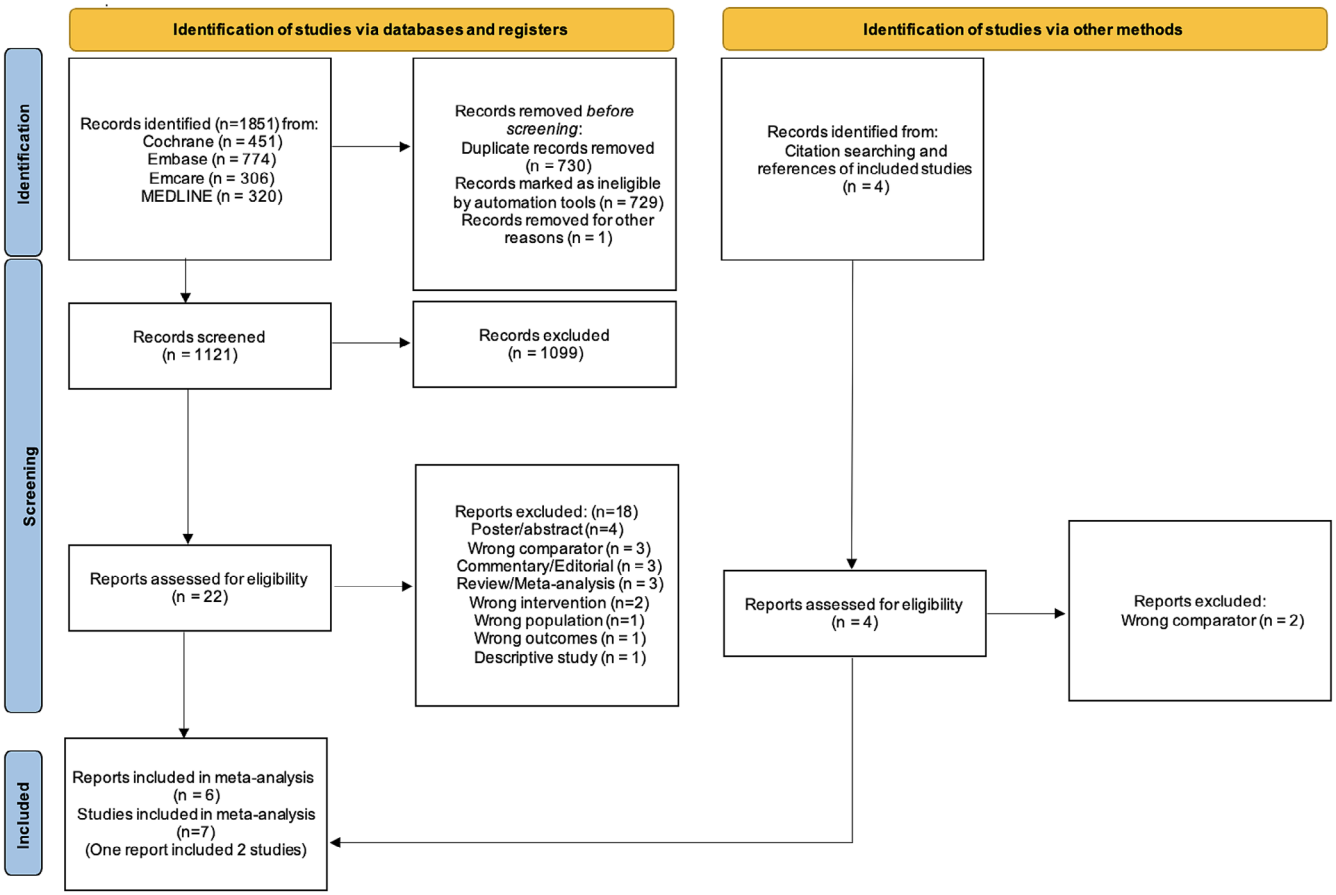
PRISMA 2020 flow diagram outlining the study selection process.

### 3.2 Study Characteristics

A total of 135 472 infants were included across seven studies: 135 149 from five observational studies [10, 18, 19, 20] and 323 from two RCTs [21, 22]. Most studies included all babies, with a mix of late preterm and term infants, although some focused on specific groups, such as elective caesarean deliveries or infants with transient tachypnea of the newborn. DR‐CPAP pressures ranged from 5 to 6 cm H_2_O, with some studies using pressures up to 8 cm H_2_O. A summary of study design, risk of bias, population characteristics, and outcomes is presented in Table 1. The details of risk of bias assessment across each study is provided in Tables S2 and S3; overall, some observational studies had concerns related to confounding [10, 19] and one RCT showed some concern due to post‐randomisation exclusions [22].

**TABLE 1:** Summary of studies included in the systematic review.

Study ID	Design	ROB	Sample size	GA (weeks)	Inclusion	Exclusion	DR‐CPAP (cm H_2_O)	Control	Outcomes evaluated
Celebi 2016 [21]	RCT	LR	259	34–38 + 6	Elective CS	PPV	5	No CPAP	1, 3, 7
Clevenger 2017 [10]	Study a: Case–control; Study b: Retrospective cohort	SC	Study a: 1019; Study b: 9483	≥ 37 + 0	All infants	PPV	5–6	No CPAP	1
Osman 2019 [22]	RCT	SC	64	34–40	TTN	Other causes of RD	5	Free flow oxygen	1, 3, 7, 8
Sandall 2025 [20]	Quality improvement	LR	23 265	≥ 36 + 0	All infants	Foetal airway anomaly	N/A	No CPAP	1
Smithhart 2019 [18, 19]^a^	Retrospective cohort	LR	6913	35–42	All infants	PPV	5–8	No CPAP	1, 2, 4
Stocks 2022 [18, 19]^a^	Retrospective cohort	SC	94 469	35–43	All infants	PPV	5–7	No CPAP	1, 2, 7

*Note:* OUTCOMES: 1. pneumothorax; 2. pneumothorax requiring intervention; 3. intubation and ventilation; 4. surfactant administration; 5. duration of respiratory support; 6. pulmonary haemorrhage; 7. nicu admission; 8. mortality.

Abbreviations: CS, caesarean section; DR‐CPAP, delivery room continuous positive airway pressure; GA, gestational age; LR, low risk; N/A, not available; PPV, positive pressure ventilation; RCT, randomised clinical trial; RD, respiratory distress; ROB: risk of bias; SC, some concerns; TTN, transient tachypnoea of newborn.

^a^ The two studies had overlapping data; for outcomes reported by both, only the study with more complete or larger data was included in the meta‐analysis. Where an outcome was reported by only one study, that study was included for that outcome.

### 3.3 Outcome Analysis

#### 3.3.1 Primary Outcome (Pneumothorax)

Meta‐analysis of unadjusted data from four studies (127 959 participants) showed a significantly increased risk of pneumothorax in the DR‐CPAP group compared to the no DR‐CPAP group (unadjusted OR: 45.79; 95% CI: 31.47–66.63; ARD: 67 more per 1000 infants; 95% CI: 47 more to 96 more), corresponding to a NNH of 15 (95% CI: 10–21) (Table 2; Figure 2) [10, 19, 20]. Similarly, analysis of adjusted data from three studies (113 435 participants) also demonstrated increased risk with DR‐CPAP (adjusted OR: 24.18; 95% CI: 2.32–251.63; ARD: 24 more per 1000 infants; 95% CI: 2–252 more; NNH = 41, 95% CI: 4–500) [10, 19]. The certainty of evidence was rated moderate for both unadjusted and adjusted data, downgraded due to risk of bias, inconsistency and potential residual confounding, despite the large effect size (Table 2; Figure 2). Meta‐analysis of the two included RCTs did not yield a pooled estimate, as there were no pneumothorax events in either of the groups [21, 22].

**TABLE 2:** Certainty of evidence assessment and summary of findings evaluating association of delivery room continuous positive airway pressure with neonatal outcomes.

Outcome	Certainty assessment							№ of patients		Effect		Certainty
	№ of studies	Study design	Risk of bias	Inconsistency	Indirectness	Imprecision	Other considerations	DR‐CPAP	No DR‐CPAP—Unadjusted	Relative (95% CI)	Absolute (95% CI)	
Observational studies (unadjusted data)												
Pneumothorax	4	Non‐randomised studies	Serious^a^	Serious^b^	Not serious	Not serious	Very strong association Residual confounding would increase the demonstrated effect	261/2716 (9.6%)	202/125243 (0.2%)	**OR: 45.79** (31.47–66.63)	**67 more per 1000** (from 47 more to 96 more)	⨁⨁⨁◯ *Moderate* ^a^, ^b^
Pneumothorax requiring interventions	1	Non‐randomised studies	Not serious	Not serious	Not serious	Very serious^c^	Residual confounding would increase the demonstrated effect	16/170 (9.4%)	15/218 (6.9%)	**OR: 1.41** (0.67–2.93)	**26 more per 1000** (from 22 fewer to 109 more)	⨁◯◯◯ *Very low* ^c^
Surfactant administration	1	Non‐randomised studies	Not serious	Not serious	Not serious	Very serious^c^	Residual confounding would increase the demonstrated effect	18/1001 (1.8%)	19/5912 (0.3%)	**OR: 5.68** (2.97–10.86)	**15 more per 1000** (from 6 more to 31 more)	⨁◯◯◯ *Very low* ^c^
NICU admission	1	Non‐randomised studies	Very serious^d^	Not serious	Not serious	Not serious	Very strong association Residual confounding would reduce the demonstrated effect	1052/1930 (54.5%)	7035/92539 (7.6%)	**OR: 14.56** (13.27–15.98)	**469 more per 1000** (from 446 more to 492 more)	⨁⨁⨁◯ *Moderate* ^d^
Observational studies (adjusted data)												
Pneumothorax	3	Non‐randomised studies	Serious^a^	Very serious^e^	Not serious	Not serious	Very strong association	−/0	−/0	**OR: 24.18** (2.32–251.63)	**24 fewer per 1000** (from 252 fewer to 2 fewer)	⨁⨁⨁◯ *Moderate* ^a^, ^e^
Randomised clinical trials												
Intubation and ventilation	2	Randomised trials	Serious^a^	Not serious	Not serious	Very serious^c^	None	0/168 (0.0%)	1/155 (0.6%)	**OR: 0.29** (0.01–7.26)	**5 fewer per 1000** (from 6 fewer to 39 more)	⨁◯◯◯ *Very low* ^a^, ^c^
NICU admission	2	Randomised trials	Serious^a^	Not serious	Not serious	Very serious^c^	None	6/168 (3.6%)	20/155 (12.9%)	**OR: 0.24** (0.09–0.63)	**95 fewer per 1000** (from 116 fewer to 44 fewer)	⨁◯◯◯ *Very low* ^a^, ^c^
Mortality	1	Randomised trials	Serious^a^	Not serious	Not serious	Very serious^c^	None	0/34 (0.0%)	1/30 (3.3%)	**OR: 0.29** (0.01–7.26)	**23 fewer per 1000** (from 33 fewer to 167 more)	⨁◯◯◯ *Very low* ^a^, ^c^

*Note:* Values represented in bold represents significant difference between groups.

Abbreviations: CI, confidence interval; NICU, neonatal intensive care unit; OR, odds ratio; RR, risk ratio.

^a^ Serious study design limitations.

^b^ Substantial heterogeneity (*I* ^2^ > 50%).

^c^ Very imprecise estimate (very few events).

^d^ Very serious study design limitations.

^e^ Considerable heterogeneity (*I* ^2^ > 75%).

**FIGURE 2:**
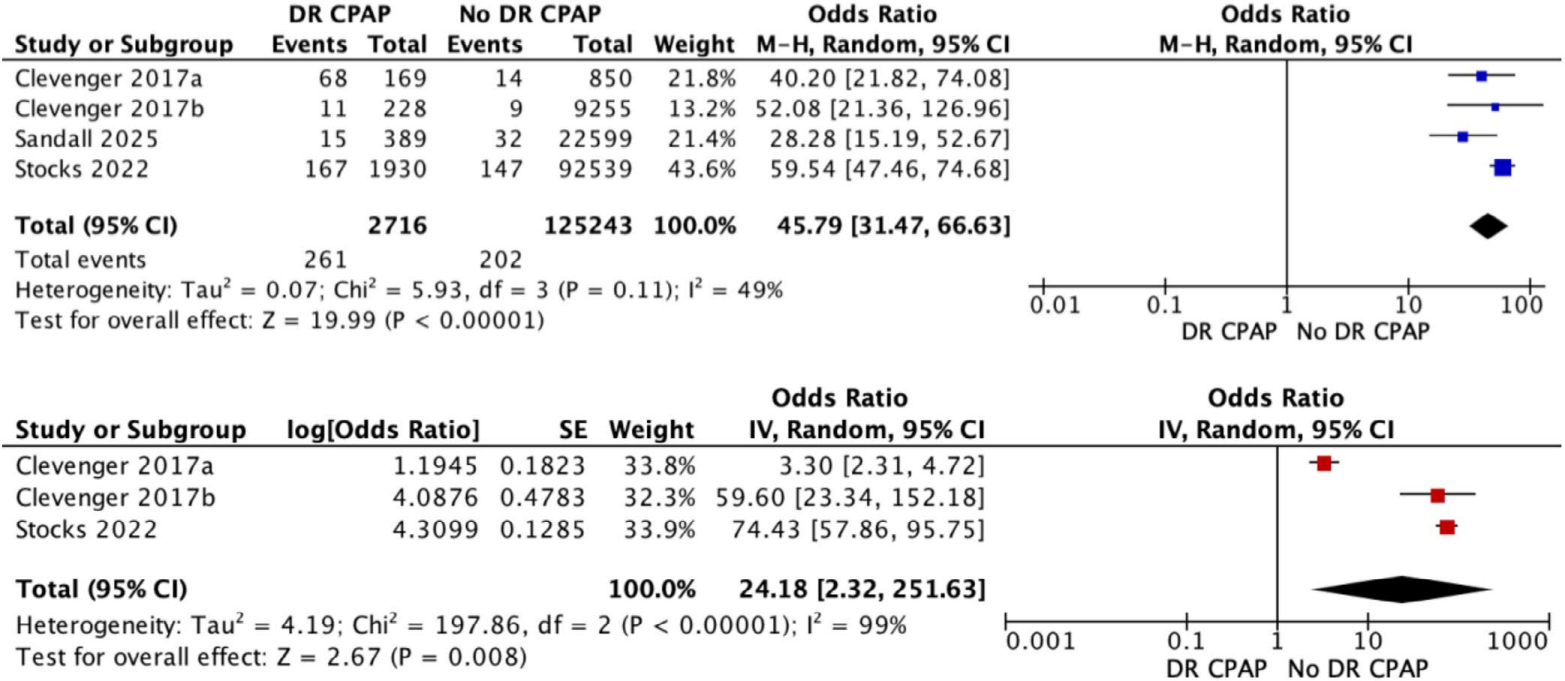
Forest plots showing the association between delivery room CPAP and pneumothorax in observational studies: (top) unadjusted data; (bottom) adjusted data.

#### 3.3.2 Secondary Outcomes

In observational studies, there was no significant difference between groups for pneumothorax requiring intervention (unadjusted OR: 1.41; 95% CI: 0.67–2.93) (Table 2; Figure S1). DR‐CPAP was associated with an increased risk of surfactant administration (unadjusted OR: 5.68; 95% CI: 2.97–10.86; 1 study, 6913 participants ARD: 15 more per 1000 infants; 95% CI: 6–31 more; NNH = 67; 95% CI: 32–167; very low certainty) (Table 2; Figure S2) [18]. Observational data also showed a significant increase in NICU admission in the DR‐CPAP group compared to those who did not receive DR‐CPAP (unadjusted OR: 14.56; 95% CI: 13.27–15.98; 1 study, 94 469 participants; ARD: 469 more per 1000 infants; 95% CI: 446–492 more; NNH = 2; 95% CI: 2–2; moderate certainty) (Table 2; Figure 3) [19].

**FIGURE 3:**
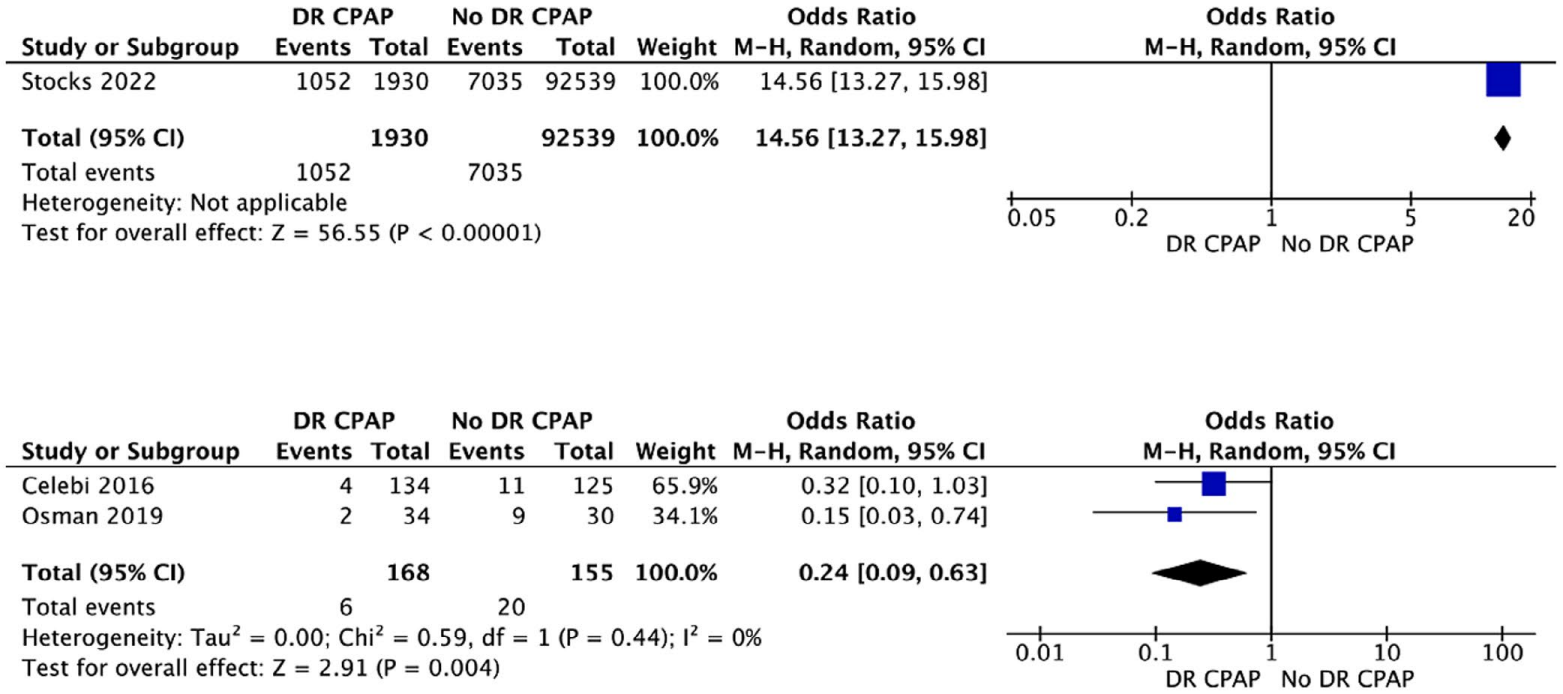
Forest plots showing the effect of delivery room CPAP on NICU admission: (top) unadjusted data from observational studies; (bottom) data from randomised controlled trials.

In contrast, the meta‐analysis of the two included RCTs showed the infants randomised to receive DR‐CPAP had a significant reduction in NICU admission (OR: 0.24; 95% CI: 0.09–0.63; 2 studies, 323 participants; ARD: 95 fewer per 1000 infants; 95% CI: 116–44 fewer; NNT = 11; 95% CI: 9–23; very low certainty) (Table 2; Figure 3) [21, 22]. RCTs also reported no significant differences between groups for intubation and ventilation (Table 2; Figure S3) or mortality (Table 2; Figure S4) [21, 22].

No data were available from either RCTs or observational studies on duration of respiratory support or pulmonary haemorrhage, and there were no separate data available to be able to perform the pre‐specified subgroup analysis.

## 4 Discussion

This systematic review and meta‐analysis included seven studies encompassing over 135 000 late preterm and term infants. Both unadjusted and adjusted analyses from observational studies showed an increased risk of pneumothorax with DR‐CPAP (*moderate certainty*). Observational data also demonstrated higher rates of NICU admission (*moderate certainty*) and surfactant administration (*very low certainty*) in infants receiving DR‐CPAP. In contrast, the two included small‐sized RCTs suggested a possible reduction in NICU admissions with DR‐CPAP (*very low certainty*), with no significant differences observed in other outcomes such as pneumothorax, intubation, or mortality (all *very low certainty*).

While the evidence suggests a possible increased risk of pneumothorax with DR‐CPAP, the certainty of these findings is moderate and should be interpreted with appropriate caution. In addition, no pneumothorax events were reported in RCTs, though these RCTs were not powered to detect such differences. However, the demonstrated association warrants consideration of how DR‐CPAP is applied in more mature infants. Unlike extremely preterm infants, who have underdeveloped muscles and limited capacity to generate negative intrapleural pressure, late preterm and term infants can produce substantial inspiratory effort to establish functional residual capacity and clear lung fluid. When this intrinsic effort is combined with external positive pressure, particularly when DR‐CPAP is initiated at 5 cm H_2_O and escalated to 8 cm H_2_O as per preterm protocols, the resulting transpulmonary pressures may become excessive, increasing the risk of air leak. This physiological interaction may help explain the observed association with pneumothorax [23]. A more selective approach, such as using DR‐CPAP only for infants with respiratory distress who require supplemental oxygen to maintain saturations, and withholding it in those with normal saturations, may help reduce the risk of pneumothorax, as suggested by a few quality improvement initiatives that reported reduced rates with such strategies [11, 18, 24].

The evidence from observational studies, which included large cohorts and were adequately powered to detect differences in outcomes like pneumothorax, contrasts with the limited data from the small, underpowered RCTs. However, interpreting observational findings requires caution due to potential confounding by indication. Infants receiving DR‐CPAP or escalating pressures on DR‐CPAP often have more severe respiratory distress and are inherently at higher risk of pneumothorax regardless of the intervention. Some studies attempted to mitigate this by adjusting for clinical markers such as delivery room oxygen use and respiratory severity, while others adjusted only for basic demographics like gestational age and sex, leaving residual confounding likely. Although randomised trials minimise such biases, detecting rare but serious events like pneumothorax [25] demands very large sample sizes and careful stratification by severity of respiratory distress or oxygen needs. Despite these challenges, the observational data offers important and compelling signals that merit further rigorous evaluation.

In contrast to the potential harms suggested by observational data, data from RCTs shows a reduction in NICU admissions with the use of DR‐CPAP. This raises the possibility that, when applied appropriately, CPAP may help facilitate lung fluid clearance and reduce the need for further respiratory support. However, these findings are based on small studies involving highly selected populations, such as infants born by elective caesarean section or those with transient tachypnea of the newborn, and may not be generalisable to the broader population of late preterm and term infants. Furthermore, the two RCTs enrolled different study populations, with one trial including only symptomatic infants requiring respiratory support and the other including all infants born by elective caesarean section, introducing clinical heterogeneity that should be considered when interpreting the pooled estimate. Nevertheless, both trials met the predefined eligibility criteria for this review and were included in the pooled analysis. It should be also noted that these trials limited CPAP to 5 cm H_2_O, a more conservative approach compared to the higher or escalating pressures used in many observational studies. This distinction suggests that low‐pressure, targeted use of DR‐CPAP in carefully chosen infants may offer benefit while avoiding the risks observed with more liberal or aggressive strategies.

### 4.1 Strengths and Limitations

This review provides a comprehensive synthesis of current evidence on the use of delivery room CPAP in late preterm and term infants, drawing from both randomised controlled trials and large observational studies. It was prospectively registered and conducted using rigorous methods, including a comprehensive literature search, independent data screening and standardised risk of bias assessments. The inclusion of both unadjusted and adjusted analyses, where available, allowed for a more nuanced interpretation of observational data, while the application of GRADE methodology provided transparent and systematic evaluation of the certainty of evidence. By incorporating physiological reasoning and contrasting findings from RCTs and real‐world data, the review offers valuable insights into both potential harms and selective benefits of DR‐CPAP.

However, the findings are constrained by several important limitations. Much of the evidence base comes from observational studies that are inherently susceptible to bias and residual confounding, even when statistical adjustment was performed. The small number and limited size of available RCTs, mostly involving highly selected populations and conservative CPAP strategies, restricts generalisability and limits confidence in pooled estimates. In addition, there was a general lack of data on secondary outcomes, with limited event rates reported, and key outcomes such as duration of respiratory support and pulmonary haemorrhage were not addressed in the included studies. The absence of granular data also precluded planned subgroup analyses, preventing exploration of heterogeneity in effect across gestational age, delivery mode, respiratory distress severity or CPAP pressure levels. Overall, the evidence was rated as low to moderate certainty, making it difficult to draw firm clinical conclusions and highlighting the need for large, well‐designed studies to guide safe and effective use of DR‐CPAP in this population.

## 5 Conclusion

In summary, this systematic review highlights the complexity of applying DR‐CPAP in late preterm and term infants, with observational data suggesting a potential increase in adverse outcomes, while small RCTs indicate a possible benefit when CPAP is used conservatively in selected populations. Nonetheless, the findings underscore the need for a more cautious and individualised approach to DR‐CPAP in this group, avoiding routine or aggressive application of high pressures. Well‐designed, adequately powered trials are needed to determine how best to use DR‐CPAP in late preterm and term infants; however, there are currently no ongoing clinical trials addressing these important uncertainties. Future studies should focus on defining optimal pressure levels, escalation strategies and comparison with other non‐invasive supports such as supplemental oxygen or high‐flow therapy.

## Author Contributions

**Yoxin Chin:** conception and design, acquisition, analysis, interpretation of data, and drafting the manuscript. **Maclean Hill:** conception and design, acquisition, interpretation of data, and revision of the manuscript. **Arun Sett:** conception and design, interpretation of data, critical revision of the manuscript for important intellectual content, supervision. **Niranjan Thomas:** conception and design, interpretation of data, critical revision of the manuscript for important intellectual content, supervision. **Abdul Razak:** conception and design, interpretation of data, critical revision of the manuscript for important intellectual content, supervision.

## Funding

No specific funding was sought for this project. Dr. Razak receives a doctoral scholarship from Monash University and the Lions Cord Blood Foundation, and also the Eric Burnard Fellowship from The Royal Australian College of Physicians. The funding body played no role in the design of the study and collection, analysis, and interpretation of data and in writing the manuscript.

## Disclosure

The protocol was registered with The Open Science Framework Registry (https://osf.io/fj8pe/).

## Ethics Statement

This study is a systematic review of previously published data and does not involve direct human participant research. Therefore, ethical approval was not required. The review was conducted in accordance with applicable guidelines and regulations.

## Conflicts of Interest

The authors declare no conflicts of interest.

## Supporting information


**Table S1:** List of excluded studies.
**Table S2:** Risk of bias for observational studies using ROBINS‐E.
**Table S3:** Risk of bias for randomised controlled trials using RoB‐2.
**Figure S1:** Forest plot of the effect of delivery room cpap on pneumothorax requiring intervention (observational data).
**Figure S2:** Forest plot of the effect of delivery room cpap on surfactant administration (observational data).
**Figure S3:** Forest plot of the effect of delivery room cpap on intubation and ventilation (randomised controlled trials).
**Figure S4:** Forest plot of the effect of delivery room cpap on mortality (randomised controlled trials).

## Data Availability

The data that supports the findings of this study are available in the Supporting Information of this article.
