# Supplementary material for: Association Between Delivery Room Continuous Positive Airway Pressure and Neonatal Outcomes in Late Preterm and Term Infants: A Systematic Review and Meta‐Analysis
Source: J Paediatr Child Health. 2026 Jul 28;62(9):1585–94. doi: 10.1111/jpc.70516 (PMC13617217; doi:10.1111/jpc.70516)
Supplement: Supplementary file 1 [file JPC-62-1585-s001.docx]

**SUPPLEMENTAL INFORMATION**

**Table of Contents**

| **Item** | **Content** | **Pages** |
| --- | --- | --- |
| 1 | Search strategies | 2-3 |
| 2 | Screening form | 4 |
| 3 | e-Table 1: List of excluded studies |  |
| 4 | e-Table 2: Risk of bias for observational studies using ROBINS-E | 5-7 |
| 5 | e-Table 3: Risk of bias for randomised controlled trials using RoB-2 | 8-10 |
| 6 | e-Figure 1: Forest plot of the effect of delivery room cpap on pneumothorax requiring intervention (observational data) | 11 |
| 7 | e-Figure 2: Forest plot of the effect of delivery room cpap on surfactant administration (observational data) | 11 |
| 8 | e-Figure 1: Forest plot of the effect of delivery room cpap on intubation and ventilation (randomised controlled trials) | 11 |
| 9 | e-Figure 2: Forest plot of the effect of delivery room cpap on mortality (randomised controlled trials) | 11 |

**SEARCH STRATEGIES**

**Medline (Ovid) Search Strategies**

1	exp Infant, Premature/ or exp Infant, Postmature/ or Term Birth/

2	((late or postmature or post-mature or post mature or preterm or pre-term or pre term or prematur* or term or fullterm* or full-term*) adj5 (baby or babies or infant* or neonat* or neo-nat* or newborn* or new-born* or new born* or birth* or week* or gestation*)).mp.

3	(("34" or thirty-four or "35" or thirty-five or "36" or thirty-six or "37" or thirty-seven or "38" or thirty-eight or "39" or thirty-nine or "40" or forty or "41" or forty-one or "42" or forty-two) adj5 (week* or gestation*)).mp.

4	((">" or "<" or "=" or ">=" or ">/=") adj5 (week* or gestation*)).mp.

5	(late preterm or late pre-term or late pre term or at-term or at term).af.

6	Gestational Age/

7	(gestational age or "GA").mp.

8	1 or 2 or 3 or 4 or 5 or 6 or 7

9	Continuous Positive Airway Pressure/

10	(continuous positive airway pressure or "CPAP" or "CPAP*").af.

11	9 or 10

12	Pneumothorax/

13	(pneumothorax or pneumothorax-associated or pneumothoraces or "air leak*").af.

14	12 or 13

15	8 and 11 and 14 (320)

**Emcare (Ovid) Search Strategies**

1	prematurity/ or postmaturity/ or term birth/

2	((late or postmature or post-mature or post mature or preterm or pre-term or pre term or prematur* or term or fullterm* or full-term*) adj5 (baby or babies or infant* or neonat* or neo-nat* or newborn* or new-born* or new born* or birth* or week* or gestation*)).mp.

3	(("34" or thirty-four or "35" or thirty-five or "36" or thirty-six or "37" or thirty-seven or "38" or thirty-eight or "39" or thirty-nine or "40" or forty or "41" or forty-one or "42" or forty-two) adj5 (week* or gestation*)).mp.

4	(late preterm or late pre-term or late pre term or at-term or at term).af.

5	gestational age/

6	(gestational age or "GA").mp.

7	1 or 2 or 3 or 4 or 5 or 6

8	continuous positive airway pressure/

9	(continuous positive airway pressure or "CPAP" or "CPAP*").af.

10	8 or 9

11	pneumothorax/

12	(pneumothorax or pneumothorax-associated or pneumothoraces or "air leak*").af.

13	11 or 12

14	7 and 10 and 13 (306)

**Embase (Ovid) Search Strategies**

1	prematurity/ or postmaturity/ or term birth/

2	((late or postmature or post-mature or post mature or preterm or pre-term or pre term or prematur* or term or fullterm* or full-term*) adj5 (baby or babies or infant* or neonat* or neo-nat* or newborn* or new-born* or new born* or birth* or week* or gestation*)).mp.

3	(("34" or thirty-four or "35" or thirty-five or "36" or thirty-six or "37" or thirty-seven or "38" or thirty-eight or "39" or thirty-nine or "40" or forty or "41" or forty-one or "42" or forty-two) adj5 (week* or gestation*)).mp.

4	((">" or "<" or "=" or ">=" or ">/=") adj5 (week* or gestation*)).mp.

5	(late preterm or late pre-term or late pre term or at-term or at term).af.

6	gestational age/

7	(gestational age or "GA").mp.

8	1 or 2 or 3 or 4 or 5 or 6 or 7

9	continuous positive airway pressure/

10	(continuous positive airway pressure or "CPAP" or "CPAP*").af.

11	9 or 10

12	pneumothorax/

13	(pneumothorax or pneumothorax-associated or pneumothoraces or "air leak*").af.

14	12 or 13

15	8 and 11 and 14 (774)

**Cochrane Search Strategies**

#1	MeSH descriptor: [Infant, Premature] explode all trees

#2	MeSH descriptor: [Infant, Postmature] explode all trees

#3	MeSH descriptor: [Term Birth] this term only

#4	((late OR postmature OR post-mature OR post mature OR preterm OR pre-term OR pre term OR prematur* OR term OR fullterm* OR full-term*) AND (baby OR babies OR infant* OR neonat* OR neo-nat* OR newborn* OR new-born* OR "new born" OR "new borns" OR birth* OR week* OR gestation*)):ti,ab,kw (Word variations have been searched)

#5	(("34" OR thirty-four OR "35" OR thirty-five OR "36" OR thirty-six OR "37" OR thirty-seven OR "38" OR thirty-eight OR "39" OR thirty-nine OR "40" OR forty OR "41" OR forty-one OR "42" OR forty-two) NEAR/5 (week* OR gestation*)):ti,ab,kw (Word variations have been searched)

#6	("late preterm" OR "late pre-term" OR "late pre term" OR at-term OR "at term") (Word variations have been searched)

#7	MeSH descriptor: [Gestational Age] this term only

#8	(("gestational age" OR "GA")):ti,ab,kw (Word variations have been searched)

#9	#1 OR #2 OR #3 OR #4 OR #5 OR #6 OR #7 OR #8

#10	MeSH descriptor: [Continuous Positive Airway Pressure] this term only

#11	("continuous positive airway pressure" OR "CPAP" OR CPAP-associated) (Word variations have been searched)

#12	#10 OR #11

#13	MeSH descriptor: [Pneumothorax] this term only

#14	(pneumothorax OR pneumothorax-associated OR pneumothoraces OR "air leak" OR "air leaking" OR "air leaks" OR "air leakage" OR "air leakages") (Word variations have been searched)

#15	#13 OR #14

#16. #9 AND #12 AND #15 (451)

**SCREENING FORM**

**INCLUSION: STUDY MUST MEET ALL THREE STEPS TO BE INCUDED**

**STEP 1**: Reports study outcome for patients ≥34 weeks’ gestational age

**STEP 2:** Reports comparison between two groups

- Neonates who received delivery room continuous positive airway pressure
- Neonates who did not receive delivery room continuous positive airway pressure

**STEP 3**: Must include at least one of the outcome data of the systematic review

1) Pneumothorax

2) Pneumothorax requiring intervention

3) Intubation and ventilation

4) Surfactant administration

5) Duration of respiratory support

6) Pulmonary haemorrhage

7) Neonatal intensive care admission

8) Mortality

**EXCLUSION: STUDY WILL BE EXCLUDED IF MEETS ANY ONE OF FOLLOWING**:

- Reports on neonates <34 weeks gestational age only
- Type of report: Commentaries, editorial letters, conference abstracts, oral presentations and review articles

| **eTable 1**: Studies excluded after full text-review | |
| --- | --- |
| **Study** | **Reason for exclusion** |
| Meberg 2007 | Not in English |
| Walker 2013 | Poster abstract |
| Yilmaz 2014 | Poster abstract |
| Hishikawa 2015 | Wrong comparator |
| Poets 2015 | Commentary/Editorial |
| Jiang 2016 | Wrong population |
| Parvathamma 2017 | Poster abstract |
| Kuttysankaran 2017 | Commentary/Editorial |
| Courtney 2018 | Wrong comparator |
| Thomas 2018 | Wrong intervention |
| Bjorland 2019 | Wrong comparator |
| Claassen 2019 | Commentary/Editorial |
| Filkins 2021 | Poster abstract |
| Handley 2022 | Wrong outcomes |
| Ramaswamy 2022 | Systematic Review/Meta-analysis |
| Shah 2022 | Systematic Review/Meta-analysis |
| Jhaveri 2024 | Review article |
| Shestak 2024 | Wrong intervention |

**References for studies in eTable 1:**

1. Meberg A, Greve-Isdahl M, Heier CA. [Pulmonary air-leakage in newborn infants]. Tidsskrift for den Norske Laegeforening : Tidsskrift for Praktisk Medicin, ny Raekke. 2007 Sep;127(18):2371-2373.
2. Walker J, Srinivasjoir R. Risk factors and management of pneumothorax in late preterm infants in a secondary unit. Unmatched case control study. J Paediatr Child Health 49(S2): 81-135.
3. Yilmaz M, Atasay B, Erdeve O, Alan S, Yildiz D, Cakir U, Kahvecioglu D, Akduman H, Arsan S. PO-0729 Can Transitional Delay And Transient Tachypnea Of Newborn Be Prevented By Cpap Intervention In Delivery Room?. Arch Dis Child. 2014;99(S2): A494.
4. Hishikawa K, Goishi K, Fujiwara T, Kaneshige M, Ito Y, Sago H. Pulmonary air leak associated with CPAP at term birth resuscitation. Arch Dis Child Fetal Neonatal Ed. 2015 Sep;100(5):F382-7. doi: 10.1136/archdischild-2014-307891. Epub 2015 Apr 8. PMID: 25854822.
5. Poets CF, Rudiger M. Mask CPAP during neonatal transition: too much of a good thing for some term infants? Arch Dis Child Fetal Neonatal Ed. 2015;100(5):F378-9.
6. Jiang S, Lyu Y, Ye XY, Monterrosa L, Shah PS, Lee SK. Intensity of delivery room resuscitation and neonatal outcomes in infants born at 33 to 36 weeks' gestation. J Perinatol. 2016 Feb;36(2):100-5. doi: 10.1038/jp.2015.156. Epub 2015 Nov 5. PMID: 26540242.
7. Parvathamma P, Makri V, Tector G, Webb J, Chakraborty M, Bagga G. Review of neonatal pneumothoraces in tertiary neonatal units in Wales. Eur J Pediatr. 2017;176(11):1445-558.
8. Kuttysankaran R, Athiraman N, Fenton AC, Ramaiah SM. Does the use of nasal continuous positive airway pressure increase the risk of a significant pneumothorax in late preterm and term infants? Pediatr Neonatol. 2017;58(5):469-70.
9. Courtney J, Callaghan S, Verner A. Retrospective audit: Has the use of nasal CPAP in term infants lead to an increased incidence of pneumothoraces?. Ulster Med J. 2018;87(2):136-148.
10. Thomas AN, Hagan JL, Lingappan K. Noninvasive ventilation strategies: which to choose? J Perinatol. 2018;38(5):447-50.
11. Bjorland PA, Oymar K, Ersdal HL, Rettedal SI. Incidence of newborn resuscitative interventions at birth and short-term outcomes: a regional population-based study. BMJ Paediatr Open. 2019;3(1):e000592.
12. Claassen CC, Strand ML. Understanding the Risks and Benefits of Delivery Room CPAP for Term Infants. Pediatrics. 2019;144(3).
13. Filkins A, Wertheimer FB, Ramanathan R, Biniwale M. Predictors and outcomes of late-preterm neonates needing respiratory support in the delivery room. J Investig Med. 2022;70:110-345.
14. Handley SC, Salazar EG, Greenberg LT, Foglia EE, Lorch SA, Edwards EM. Variation and Temporal Trends in Delivery Room Management of Moderate and Late Preterm Infants. Pediatrics. 2022;150(2).
15. Ramaswamy VV, Abiramalatha T, Bandyopadhyay T, Shaik NB, Pullattayil S AK, Cavallin F, Roehr CC, Trevisanuto D. Delivery room CPAP in improving outcomes of preterm neonates in low-and middle-income countries: A systematic review and network meta-analysis. Resuscitation. 2022 Jan;170:250-263. doi: 10.1016/j.resuscitation.2021.10.027. Epub 2021 Oct 29. PMID: 34757058.
16. Shah BA, Fabres JG, Leone TA, Schmolzer GM, Szyld EG, International Liaison Committee on Resuscitation Neonatal Life Support Task F. Continuous positive airway pressure for term and >/=34(+0) weeks' gestation newborns at birth: A systematic review. Resusc Plus. 2022;12:100320
17. Jhaveri V, Vali P, Giusto E, Singh Y, Lakshminrusimha S. Pneumothorax in a term newborn. J Perinatol. 2024;44(4):465-71.
18. Shestak EV, Kovtun OP, Mylarshikova EA, Nechaeva YI. Efficacy and Safety of a Standardized CPAP Protocol in the Delivery Room in Late Preterm Infants with Infectious and Non-Infectious Lung Diseases. Gen Reanimatol. 2024;20(5):44-54.

| eTable 2: Risk of Bias for Observational Studies using ROBINS-E | | | |
| --- | --- | --- | --- |
| **Study** | **Domain** | **Risk of Bias** | **Reason** |
| **Clevenger 2017** | Bias due to Confounding | Some concerns | Did not control for relevant confounders |
|  | Bias in Classification of Exposure | Low risk | Clear definition of exposure |
|  | Bias due to Selection of Participants | Low risk | Included all pre-specified infants |
|  | Bias due to Deviations from Intended Exposures | Low risk | No deviation |
|  | Bias due to Missing Data | Low risk | No missing data |
|  | Bias due to Measurement of Outcomes | Some concerns | Assessors aware of exposure history |
|  | Bias in Selection of Reported Results | Low risk | All results reported |
|  | **Overall Bias** | Some concerns |  |
| **Sandall 2025** | Bias due to Confounding | Low risk | Controlled for relevant confounders |
|  | Bias in Classification of Exposure | Low risk | Clear definition of exposure |
|  | Bias due to Selection of Participants | Low risk | Included all pre-specified infants |
|  | Bias due to Deviations from Intended Exposures | Low risk | No deviation |
|  | Bias due to Missing Data | Low risk | No missing data |
|  | Bias due to Measurement of Outcomes | Low risk | Data on event rates entered prior to project |
|  | Bias in Selection of Reported Results | Low risk | All results reported |
|  | **Overall Bias** | Low risk |  |

| eTable 2 (continue): Risk of Bias for Observational Studies using ROBINS-E | | | |
| --- | --- | --- | --- |
| **Study** | **Domain** | **Risk of Bias** | **Reason** |
| **Smithhart 2019** | Bias due to Confounding | Low risk | Logistic regression to control for confounders |
|  | Bias in Classification of Exposure | Low risk | Clear definition of exposure |
|  | Bias due to Selection of Participants | Low risk | Included all pre-specified infants |
|  | Bias due to Deviations from Intended Exposures | Low risk | No deviation |
|  | Bias due to Missing Data | Low risk | No missing data |
|  | Bias due to Measurement of Outcomes | Low risk | Pneumothorax diagnosis entered prior to study |
|  | Bias in Selection of Reported Results | Low risk | All results reported |
|  | **Overall Bias** | Low risk |  |
| **Stocks 2022** | Bias due to Confounding | Some concerns | Did not control for relevant confounders |
|  | Bias in Classification of Exposure | Low risk | Clear definition of exposure |
|  | Bias due to Selection of Participants | Low risk | Included all pre-specified infants |
|  | Bias due to Deviations from Intended Exposures | Low risk | No deviation |
|  | Bias due to Missing Data | Low risk | No missing data |
|  | Bias due to Measurement of Outcomes | Some concerns | Assessors aware of exposure history |
|  | Bias in Selection of Reported Results | Low risk | All results reported |
|  | **Overall Bias** | Some concerns |  |

| eTable 3: Risk of Bias for Randomised Controlled Trials using RoB-2 | | | |
| --- | --- | --- | --- |
| **Study** | **Domain** | **Risk of Bias** | **Reason** |
| **Celebi 2016** | Bias due to Randomisation Process | Low | Infants were randomised |
|  | Bias due to Deviations from Intended Interventions | Low | No deviation |
|  | Bias due to Missing Outcome Data | Low | No missing data |
|  | **Bias in Measurement of Outcomes** | Low | Assessors not blinded + method of measuring unspecified should this be some concerns? |
|  | Bias in Selection of Reported Results | Low | All results reported |
|  | **Overall Bias** | Low |  |
| **Osman 2019** | Bias due to Randomisation Process | Low | Computer-generated randomisation |
|  | Bias due to Deviations from Intended Interventions | Some concerns | Multiple patients excluded from analysis in both arms |
|  | Bias due to Missing Outcome Data | Some concerns | No data on patients randomized and then excluded from analysis |
|  | Bias in Measurement of Outcomes | Low | Same assessment of all patients included in study |
|  | Bias in Selection of Reported Results | Low | All results reported |
|  | **Overall Bias** | Some concerns |  |

e-Figure 1: Forest plot of the effect of delivery room CPAP on pneumothorax requiring intervention (observational data)

e-Figure 2: Forest plot of the effect of delivery room CPAP on surfactant administration (observational data)

e-Figure 3: Forest plot of the effect of delivery room CPAP on intubation and ventilation (randomised controlled trials)

e-Figure 4: Forest plot of the effect of delivery room CPAP on mortality (randomised controlled trials)
